# Posterior stabilization and sub-total pectoralis major release for combined dynamic functional and structural posterior instability: a case report

**DOI:** 10.1016/j.xrrt.2026.100861

**Published:** 2026-08-22

**Authors:** Alahan Alkis Sensoy, Muhammed Yusuf Afacan, Tobias Gruber, Doruk Akgün, Alp Paksoy

**Affiliations:** a Center for Musculoskeletal Surgery, Charité-Universitätsmedizin Berlin, Corporate Member of Freie Universität Berlin and Humboldt-Universität zu Berlin, Berlin, Germany; b Istanbul Physical Therapy and Rehabilitation Training and Research Hospital, Department of Orthopaedics and Traumatology, Istanbul, Turkiye; c Istanbul University-Cerrahpasa, Institute of Graduate Studies, Cerrahpasa Faculty of Medicine, Department of Anatomy, Istanbul, Turkiye; d Center for Orthopedic and Trauma Surgery, University Medical Center of Cologne, Cologne, Germany

## Introduction

Understanding the patho-mechanism of posterior shoulder instability (PSI) is a decisive step in planning individualized therapy.^6^ To guide diagnosis and treatment, the ABC classification^22^ categorizes PSI into first-time (type A), dynamic (type B), and static (type C). Type B of the ABC classification is further divided into functional (B1) and structural (B2) dynamic instability. The B1 sub-type is characterized by a pattern in which the instability is caused by pathologic activation of the rotator cuff and peri-scapular muscles.^7^^,^^10^ In addition to rotator cuff muscles, the pectoralis major stabilizes the glenohumeral joint by resisting superior migration of the humeral head^5^^,^^23^ and enhancing scapulo-thoracic stabilization of the latissimus dorsi and deltoid muscles.^2^ Dysfunction of the pectoralis major may compromise glenohumeral stability^15^; however, existing literature primarily associates its abnormalities with anterior^1^^,^^10^^,^^13^^,^^15^, ^16^, ^17^^,^^24^ or multi-directional^3^ shoulder instability.

Historically, the absence of a structural pathology on diagnostic imaging, coupled with the limited success of conventional treatments, frequently led to the dismissal of this sub-type of PSI as an attention-seeking or a psychiatric behavior.^8^^,^^18^^,^^19^ This misconception not only delayed appropriate care but also increased the disease burden and social stigmatization for affected patients.^21^ Furthermore, the previously proposed strategy of “skillful neglect”^9^ has been challenged because of the severe subjective suffering and functional limitations experienced by patients.^19^^,^^21^ Surgical stabilization is generally not recommended for dynamic-functional instability, as it is associated with poor outcomes.^19^^,^^22^^,^^26^ Consequently, the current gold standard focuses on normalizing this pathologic muscle-activation pattern^20^^,^^26^ through specialized physiotherapy,^19^ as well as the use of a shoulder pacemaker.^21^ However, a B1 PSI may cause structural defects after repetitive dislocation events and create a combination of B1/B2 instability, which may necessitate a combined treatment of B1 and B2 instability.

To the best of current knowledge, no clinical data exist on the relationship between pectoralis major hyperactivity and PSI, nor on surgical release of the pectoralis major as a treatment option for the B1 sub-type. This case is best interpreted either as a rare form of type-B1 posterior instability with associated structural lesions after recurrent dynamic posterior dislocation events or as a transitional pattern with overlapping B1/B2 characteristics.

### Case report

#### Pre-operative assessment

A 41-year-old male presented to our clinic in October 2023 with atraumatic, recurrent, uncontrolled posterior dislocations of the right shoulder for approximately two years. He reported movement-dependent pain associated with posterior shoulder dislocations, which led to avoidance behavior regarding shoulder use and a substantial subjective burden. The patient was initially treated at another hospital with a conservative treatment using a shoulder pacemaker for eight weeks, but without symptom relief. During the clinical examination, the patient demonstrated a positive Show-me test. The patient avoided forward flexion of the shoulder and abduction beyond 90° because these movements, particularly when combined with internal rotation, triggered involuntary postero-superior subluxation of the humeral head with spontaneous relocation (Video 1, *a*). The remaining range of motion was unrestricted. In addition, the patient exhibited scapular dyskinesis, identified through visual combined palpation according to Kibler's method,^12^ and the scapular assistance test was positive meaning that it was able to stabilize the glenohumeral joint (Video 1, *b*).^11^ Interestingly, the shoulder was also stabilized when manual inhibition of the pectoralis major muscle was applied (Video 1*c*).

The patient underwent computed tomography (Video 2, *a-c*) and magnetic resonance imaging (Video 3, *a-c*) of the right shoulder. The computed tomography scan revealed a minimal osseous involvement without a relevant posterior bony defect, whereas the magnetic resonance imaging scan showed labral fissuring without displacement. Owing to the noticeable lateral scapular winging, electromyography (EMG) and nerve conduction studies (NCS) were performed to assess the following muscles bilaterally to compare pathologic to the healthy side; supraspinatus, infraspinatus, teres minor, serratus anterior, trapezius, pectoralis major, latissimus dorsi, and deltoid. EMG/NCS revealed normal activity in all measured muscles for both sides except resting hyperactivity of the pectoralis major muscle compared to the healthy left side. The combination of the EMG result and the positive clinical test with manual inhibition of the pectoralis major muscle was considered as supporting the hypothesis that pectoralis major overactivity contributed to the instability.

Given the failure of conservative treatments and the high level of suffering, the option of surgical intervention was discussed with the patient. The planned procedure included arthroscopic posterior stabilization combined with an open sub-total release of the pectoralis major muscle.

### Surgical procedure

**Figure 1:**
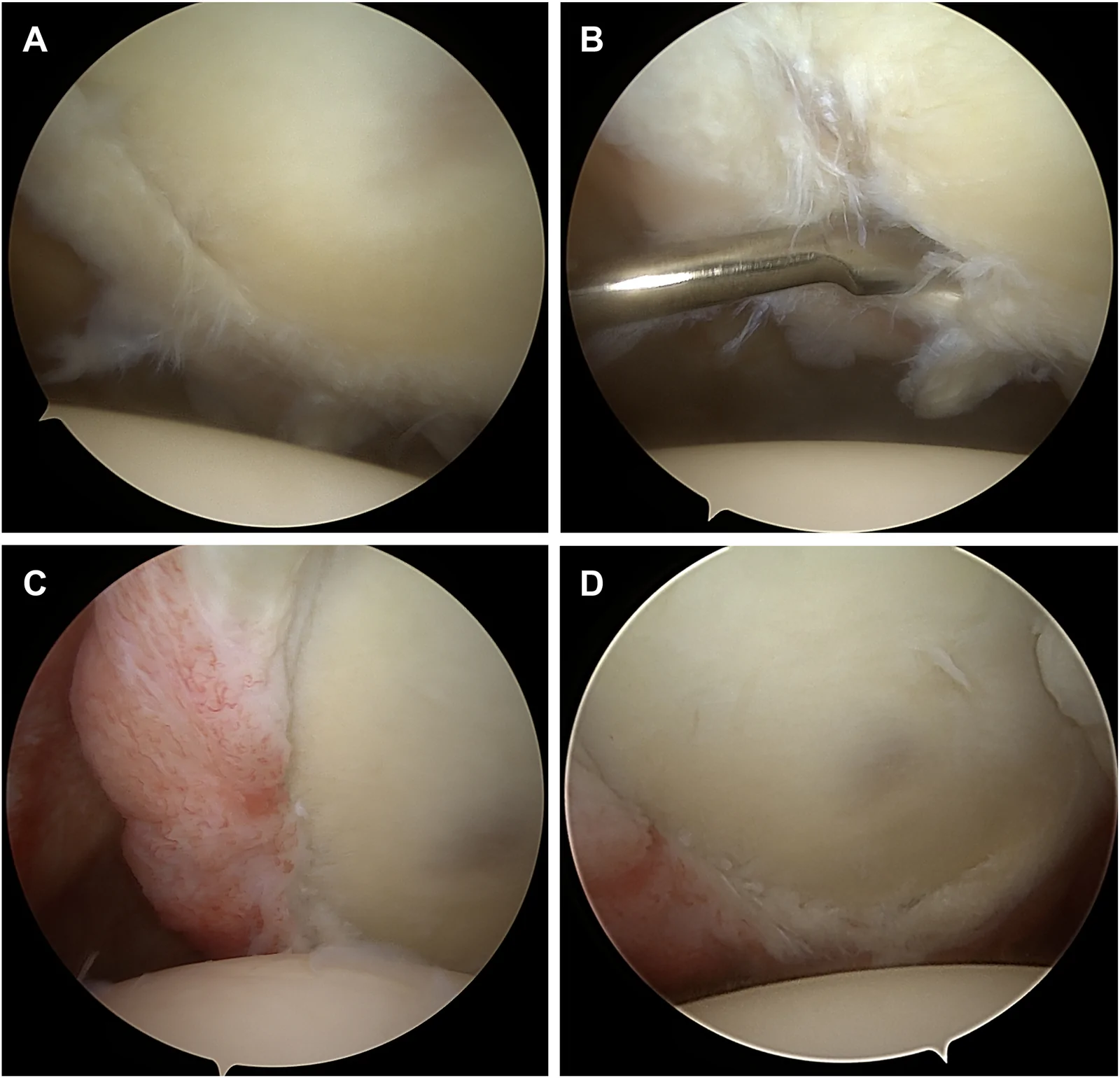
Arthroscopic images depicting the procedure. (**a** and **b**) Posterior labral fissuring of the posterior capsulolabral complex. (**c** and **d**) Postero-inferior capsulolabral bump after anchor repair.

The surgery was performed under general anesthesia in the beach chair position and began arthroscopically, starting with the establishment of a posterior viewing portal. A diagnostic arthroscopy was conducted using a 30° scope. A posterior Bankart lesion was identified (Fig. 1, *A* and *B*), and the glenoid as well as the superior labrum were intact. The rotator cuff muscles appeared without pathologic findings. No reverse Hill-Sachs lesion was detected. During diagnostic arthroscopy, the intra-articular portion of the long head of the biceps tendon appeared intact and without pathology. Nevertheless, it was tenotomized by electrocautery to allow subsequent open biceps tenodesis during the anterior approach. This was done to reduce the risk of secondary biceps instability or tendon-related symptoms after pectoralis major release.

An antero-inferior portal and a transtendinous antero-superior portal were then established. The optic was repositioned antero-superiorly. Two transparent twist-in cannulas were inserted into the anterior and posterior portals. The posterior and postero-inferior parts of the labrum were mobilized from the scapular neck to restore its mobility. Following this, an additional postero-lateral portal was created bluntly, with strict attention to preserving the axillary nerve. Starting at the 6:30 position, 5 1.8-mm all-suture anchors (FiberTak, Arthrex, Naples, FL, USA) were placed in sequence. Both suture ends were passed through the posterior capsulolabral complex to create extra-articular mattress sutures. Through this procedure, the posterior labrum was anatomically re-fixed onto the glenoid rim, and the postero-inferior capsule was tightened in a postero-inferior-to-superior capsular shift, resulting in good tension of the postero-caudal capsular structures (Fig. 1, *C* and *D*). The instruments were then removed, and the arthroscopy portals closed.

**Figure 2:**
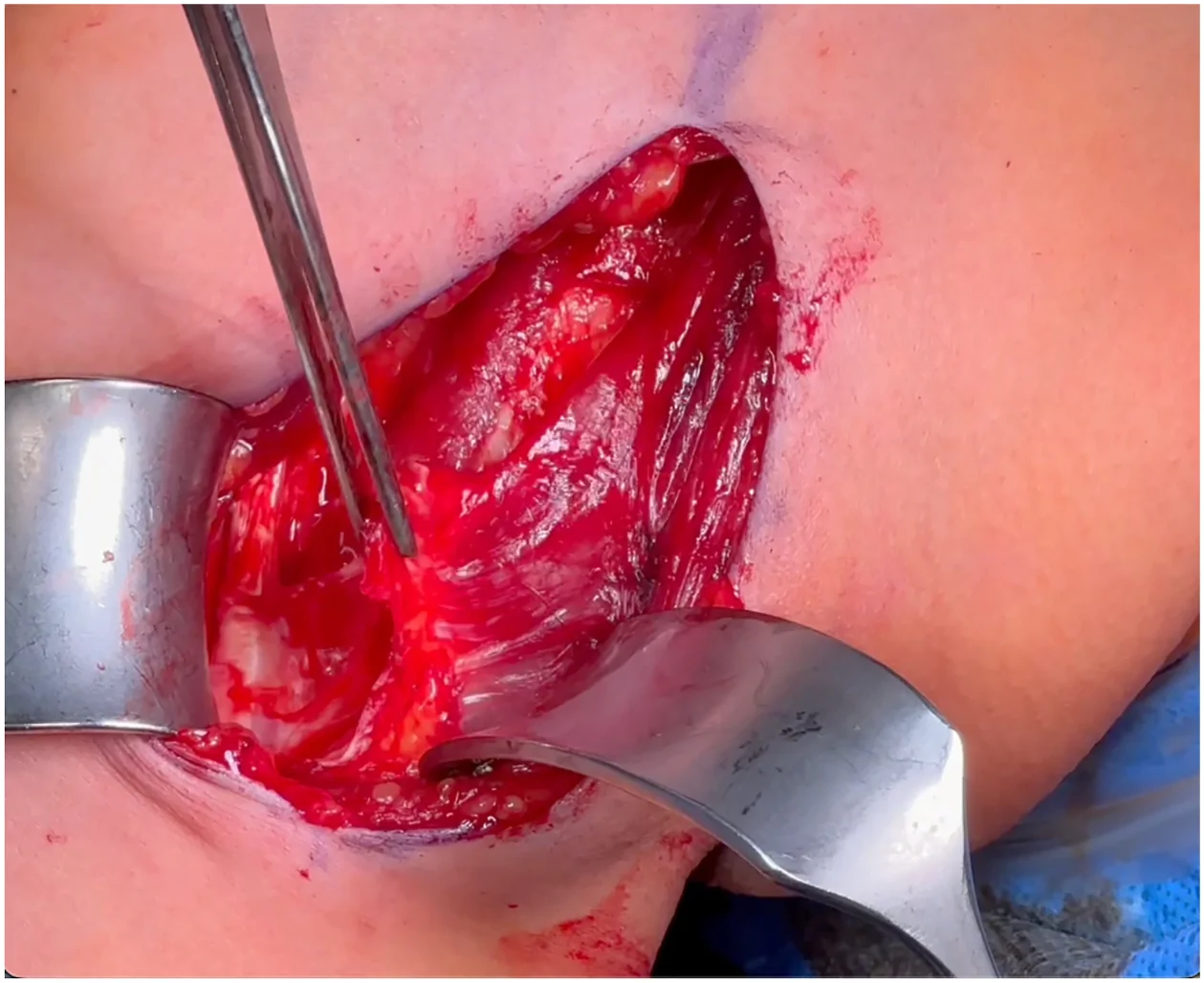
Released sternocostal head of the pectoralis major.

A deltopectoral approach was performed. After identifying the pectoralis major tendon, the sternocostal head was released, while its clavicular head was left in its anatomic position (Fig. 2 and Video 4). As the proximal part of the long head of the biceps tendon had already been released arthroscopically, an open sub-pectoral biceps tenodesis was then performed distal to the pectoralis major insertion using a suture anchor to minimize the risk of secondary biceps-related instability or symptoms. A layered closure of the wound was conducted, and a sterile wound dressing was applied. Finally, an external rotation orthosis was applied.

### Post-operative rehabilitation

The patient was immobilized post-operatively with an external rotation orthosis for six weeks.

The shoulder was mobilized passively for the first four weeks with flexion up to 45°, abduction up to 45°, and external rotation up to 30°, whereas no internal rotation was allowed. In the following two weeks, the passive range of motion was increased up to 90° of flexion, abduction up to 70°, and external rotation up to 50°, while internal rotation remained restricted at 0°. From the seventh week onward, full flexion and abduction were progressively restored. Internal rotation exercises were initiated, and active mobilization was increasingly encouraged. After 12 weeks, sport-specific training was allowed.

### Follow-up evaluations

At the 1-year follow-up, the patient was pain-free, with unrestricted shoulder function, and no episodes of dislocation had been reported since the surgery. The patient had a forward flexion of 170°, abduction of 170°, external rotation of 70°, and an internal rotation behind the back up to T12 (Video 5). No signs of instability or dislocation were observed, either in full flexion or in full abduction.

## Discussion

PSI is a complex and multi-factorial condition, and the dynamic functional sub-type B1 presents therapeutic challenges because instability arises primarily from aberrant muscle activation rather than structural defects. According to the treatment algorithm proposed by Moroder et al,^21^^,^^22^ the primary management for type-B1 instability consists of intensive physiotherapy focused on scapular coordination and external rotator activation, potentially augmented by a shoulder pacemaker to normalize muscle patterns.^21^, ^22^, ^26^ Surgical stabilization is generally unwarranted in this group because historical data indicate unpredictable results in this specific cohort^19^ with high recurrence rates, potential worsening of pain, and movement restriction^22^^,^^26^; however, it has been noted that surgical treatment of evident structural deficiencies may be attempted as a salvage procedure if instability persists despite comprehensive functional therapy.^22^ Furthermore, a B1 PSI can rarely cause structural defects after repetitive dislocation events and create a combination of B1/B2 instability. The treatment should therefore address the structural as well as functional causes of this combined pathology. In line with this concept, we additionally performed a prophylactic biceps tenodesis after an arthroscopic tenotomy and anterior exposure, aiming to minimize the risk of secondary biceps-related instability or symptoms.

In the present case, the patient remained symptomatic despite strict adherence to these recommended protocols, including shoulder pacemaker therapy. Moroder et al^18^^,^^21^ described functional instability as resulting from pathologic activation of large shoulder muscles with underactivity of stabilizers and demonstrated that neuromodulatory approaches such as the use of a shoulder pacemaker can restore stability in selected patients. Maybe this is the reason why the shoulder pacemaker did not help this patient, as no hypoactivity especially of the external rotators was detected in EMG but a hyperactivity of the pectoralis major which cannot be intervened with shoulder pacemaker treatment. It should be emphasized that EMG/NCS examinations should include almost all the shoulder girdle muscles and should be compared to the examinations of a contralateral healthy shoulder in order to find the underlying pathology, as any muscle of the shoulder girdle can cause instability.

Kowalski et al^14^ showed that the pectoralis major demonstrates high activation amplitudes during unstable push-up variations, illustrating its capacity to generate substantial internal rotation and adduction forces. Although these findings are from exercise studies, they demonstrate that the pectoralis major can dominate shoulder kinetics under load. This mechanism mirrors the patho-mechanics also seen in sequelae of obstetrical brachial plexus palsy, where internal rotation contractures lead to posterior dislocations.^4^

In contrast to these biomechanical insights, the existing clinical literature on B1 sub-type of PSI remains focused on other muscle groups. While functional shoulder instability has been characterized by pathologic activation patterns of the rotator cuff and peri-scapular muscles (eg, latissimus dorsi, pectoralis minor), the existing diagnostic and therapeutic considerations do not identify pectoralis major overactivity as a principal driver.^10^^,^^18^^,^^21^ According to the dynamic EMG findings reported by Jaggi et al,^10^ heightened activation of the latissimus dorsi appears to contribute to both anterior and posterior instability patterns, whereas elevated activity of the pectoralis major seems more closely associated with anterior instability. Similarly, in the study conducted by Page,^25^ it was highlighted that anterior muscle tightness/imbalance (including pectoralis tightness) alters scapulo-humeral mechanics, functionally reducing sub-acromial clearance and contributing to anterior-biased symptoms. The biomechanical review by Bäcker et al^2^ emphasized the role of the pectoralis major in contributing to scapulo-thoracic stability through force coupling with the latissimus dorsi and deltoid but did not report evidence of pectoralis major hyperactivity as a cause of posterior translation. Taken together, these studies highlight the pectoralis major as an important dynamic stabilizer of the shoulder, whereas the present case uniquely identifies it as a pathologic contributor to the B1 sub-type of PSI. Interestingly, the effect of the pectoralis major can be clinically tested through manual de-activation as seen in this patient, which resulted in a stable glenohumeral joint (Video 1, *b*). Furthermore, it is important to look for structural co-injuries even in B1 patients, as these can occur due to repetitive dislocations and should also be addressed in a surgical setting.

## Conclusion

This case expands both the mechanistic and therapeutic understanding of dynamic functional PSI by demonstrating that resting hyperactivity of the pectoralis major can serve as a primary and potentially modifiable driver. In these cases, targeted release of the sternocostal head of the pectoralis major in combination with posterior stabilization may restore balance and stability in short-term function. Incorporating shoulder girdle muscle activity assessment into the diagnostic algorithm in B1 PSI may ultimately allow for more individualized treatment strategies in this challenging patient population.

## Disclaimers:

Funding: No funding was received for this work.

Conflicts of interest: The authors, their immediate families, and any research foundation with which they are affiliated have not received any financial payments or other benefits from any commercial entity related to the subject of this article.

Patient consent: Written informed consent for publication of this case report and the accompanying images and videos was obtained from the patient.

## Supplementary Data

Video 1a Clinical examination.

Video 1b Stabilization of the joint through scapular assistance test.

Video 1c Stabilization of the joint through manual de-activation of the pectoralis major.

Video 2a Pre-operative computed tomography. Multi-planar reconstructions (axial, coronal, and sagittal) demonstrating a small bony reaction at the posterior glenoid rim without relevant bony insufficiency.

Video 2c

Video 3a Pre-operative magnetic resonance imaging. Multi-planar reconstructions (axial, coronal, and sagittal) demonstrating a posterior Bankart lesion.

Video 3c

Video 4 Open surgical procedure showing the release of the sternocostal head of the pectoralis major.

Video 5 Clinical examination at 1-year follow-up demonstrating full range of motion without recurrent instability.
